# Are meat options preferred to comparable vegetarian options? An experimental study

**DOI:** 10.1186/s13104-021-05451-9

**Published:** 2021-01-26

**Authors:** Rachel Pechey, Gareth J. Hollands, Theresa M. Marteau

**Affiliations:** 1 Behaviour and Health Research Unit, Institute of Public Health, University of Cambridge, Cambridge, UK; 2 Nuffield Department of Primary Care Health Sciences, University of Oxford, Oxford, OX2 6GG UK

**Keywords:** Meat, Vegetarian, Preferences, Food, Sustainability, Health

## Abstract

**Objective:**

Reducing meat consumption would have substantial benefits both in terms of health and environmental impact, but meat options may be more attractive to customers than meat-free options. This study tested this by presenting UK adults (n = 540) with a series of pictures showing two meal options and asking them to select which they would prefer to eat right now. They completed this task for every possible pair from a pool of six comparator meat-based options and six target options (66 pairs). Participants all saw identical comparator options, and were randomised to see the same pictures of target options but with descriptions that suggested they were either meat-based or vegetarian.

**Results:**

Selections were used to rank the options for each individual from 1 (most-selected) to 12 (least-selected). Vegetarian target options were ranked worse [by 1.23 places (95% CI: 1.02, 1.44)] than meat target options. Higher self-reported consumption of meat predicted worse mean rankings of target options when these were vegetarian, but not when target options were meat-based. This suggests meat options are preferred to equivalent vegetarian options and may be more likely to be selected. This has implications for interventions aiming to reduce meat consumption to make diets healthier and more sustainable.

## Introduction

Reducing meat consumption could lead to substantial benefits both in terms of health and environmental impact [1]. However, one barrier to changing meat consumption may be societal and individual preferences for eating meat—with meat consumption described as being natural, normal, necessary and nice [2]. Preferences for meat may play a substantial role in determining food selection and consumption, with the most common reason given for eating meat in a large Belgian survey being taste [3]. This reflects reported barriers to reducing meat consumption including enjoying meat and family food preferences [4]. Moreover, those with stronger preferences for meat may be most resistant to changing their behaviour [5]. In contrast, meat substitutes may have limited acceptance given their unfamiliarity and perceived lower sensory attractiveness [6].

Given the significance placed on preferences in self-reported motivations for selecting foods that contain meat, the current study aimed to experimentally examine the extent to which preferences differ depending on whether the available options are meat-based or vegetarian. In particular, in order to try to isolate differences due to the presence or absence of meat, the study compared responses to the same picture of a meal, which was described as either a vegetarian option or an equivalent option containing meat. This tightly-controlled study allows an exploration of the extent to which the meat component drives preferences, independent of the meal type and the visual attractiveness of the meal.

## Main text

### Methods

#### Participants

A sample of 540 UK adults was recruited from existing members of a market research agency panel (Dynata), Participants were invited to participate via email sent by Dynata, or links placed on their website. Quotas were set to obtain equal numbers by highest educational qualification (lower: Up to GCSE level or 1 A level; higher: 2 + A levels or equivalent, or higher qualification), and to ensure a representative sample by age and gender. Participants with dietary restrictions (e.g., vegetarians) were excluded, to ensure that participants could select any of the options offered. Quality checks included excluding participants who failed attention check questions (n = 87) or completed the study in < 30% of the median time (n = 1).

The sample size was based on a t-test to find an effect size of d = 0.28 (equivalent to the difference in preference rankings between lower energy and higher energy main meals in a previous study, due to there being no direct evidence and lower energy options in this previous study including more vegetarian options [7]), with alpha = 0.05, and power of 0.9. The sample size required was calculated using G*Power 3.1.9.2 to be 270 per group, so 540 in total.

#### Design

This was an online study, with one between-subjects factor (meat vs. vegetarian options).

The study was pre-registered on the Open Science Framework (https://osf.io/yjmpe) and ISRCTN (http://www.isrctn.com/ISRCTN15043170). Ethical approval was obtained from the University of Cambridge Psychology Research Ethics Committee (Ref: Pre.2020.030).

A second aim of this study was to act as a pilot study to identify options to be used in a subsequent study—this aim has been written up elsewhere [7].

#### Materials

Six comparator and six target main meal options were identified from the manual used in a previous study [7]. Due to the second aim of this study (acting as a pilot for a subsequent study), comparator meal options were all higher energy, while target options were lower energy (defined as those with under 500 kcal for a complete meal, whereas higher energy had 500 kcal or more [8]).

All the comparator meal options were meat-based (see Table 1 for the list of options). Meat and vegetarian versions were created for target meal options, which were described under the same dish name, aside from the meat vs. vegetarian content (e.g., vegetable Balti vs. chicken Balti). The same photograph was used for both the meat and vegetarian versions of the target options, with the dish name displayed underneath.

**Table 1:** Mean (s.d.) rankings by selection for each food option, by study condition, with higher values indicating less-selected options

		Meat condition	Vegetarian condition
**Comparator**	Lasagne (beef)	4.5 (3.2)	3.8 (3.0)
	Battered fish	4.7 (3.7)	3.9 (3.2)
	Beef pie	6.4 (3.3)	5.3 (3.3)
	Arrabbiata with meatballs	6.8 (3.3)	5.3 (3.1)
	BBQ Chicken	7.0 (3.0)	5.6 (3.0)
	Chicken Milanese	7.6 (3.0)	6.0 (2.9)
	Mean	*6.2* (*1.1*)	*5.0* (*1.3*)
**Target**	Cottage pie (beef/soya)	5.4 (3.5)	7.2 (3.2)
	Fajita (chicken/vegetable)	5.9 (3.6)	7.9 (3.1)
	Balti (chicken/vegetable)	6.9 (3.2)	7.8 (2.8)
	Black bean (chicken/tofu)	7.2 (3.2)	9.6 (2.8)
	Cajun (beef/vegetable)	7.6 (2.8)	7.7 (2.5)
	Chilli (turkey/soya)	7.9 (3.3)	7.8 (2.8)
	Mean	*6.8* (*1.1*)	*8.0* (*1.3*)

N.B. Rankings for individual options go from 1 (most-selected from paired-selections) to 12 (least-selected). Mean rankings for the target and comparator categories are bounded at 3.5 (all six options belonging to that category are ranked 1–6—the top six places) and 9.5 (all six options belonging to that category are ranked 7–12—the bottom six places)

#### Procedure

Each participant was randomised to see target options that were labelled as either meat-based or vegetarian options. Participants all saw the same six comparator meal options, which were all meat-based.

Participants were presented with pictures of two food options, and asked to select which they would prefer to eat right now. They completed this task for every possible item pair for the comparator main meal options and their assigned target options (66 item pairs).

Participants then completed measures of age, gender, highest educational qualification, household income, usual meat consumption (“How often do you usually eat meat?” 6 options from ‘Less than once a week’ to ‘More than twice a day’) and hunger (7 point rating scale, from ‘Very hungry’ to ‘Very full’).

#### Analyses

The primary outcome was the mean ranking for target options, based on the number of times it was selected. For each trial, the selected item received a score of 1. Scores were summed across all trials for each item for each participant. Rankings for the meal options were created for each participant, from 1 (most-selected from paired-selections) to 12 (least-selected). For ties in relative rankings both tied items’ rankings were recorded as 1.5, 2.5 or 3.5 (i.e. tied for first, second or third place, respectively). As such, higher rankings indicate options that are less-selected, implying these are less-preferred.

The primary analysis was a multiple regression predicting the rankings for target meals depending on whether these options were vegetarian or meat-based (with the reference group coded as the meat-based target options). Age, gender, education and hunger were included as covariates.

Usual meat consumption, and its interactions with meal meat content, were then added to the above model to examine whether any difference in rankings for meat-based vs. vegetarian target options was moderated by usual meat consumption.

## Results

Of the 540 participants, 51.3% were female (n = 277). The mean age was 46.9 (s.d. 16.8; range 18–79), and 50% had lower education (n = 270). The majority were white (92.6%, n = 497; 7.3% other, n = 38; 0.9% missing, n = 5), and the mean hunger rating was 0.60 (s.d. 1.38). In terms of usual meat consumption, 30.7% (n = 116) reported eating meat 3 times per week or less, 47.0% (n = 254) 4–6 times per week and 22.2% (n = 120) daily.

The distribution of participants between the meat vs. vegetarian conditions was not exactly equal, due to exclusions occurring after randomisation for speeding or failing attention checks, with 275 participants in the vegetarian condition (50.9%) and 265 in the meat condition (49.1%).

### Primary analysis

In terms of selections, target options were selected on average 31.1 times (s.d. 6.3) in the meat condition and 24.2 times (s.d. 7.3) in the vegetarian condition (means for comparator options being 34.9 times and 41.8 times, respectively).

Preferences were examined by looking at the mean rankings by selection for target and comparator options (see Table 1), with comparator options tending to be more selected but this distinction being greater in the vegetarian target options condition than the meat target options condition (mean of 6.2 for comparator options vs. 6.8 for target options in the meat condition; 5.0 vs. 8.0 in the vegetarian condition).

Examination of the data revealed that a number of data points for vegetarian options were at the maximum score (i.e., consistently avoided) (n = 36). Therefore a Tobit regression model was used. The model suggested a coefficient of 1.23 (95% CI: 1.02, 1.44; p < 0.001) for vegetarian (rather than meat) options (controlling for age, gender, education and hunger) (see Additional file 1: Table S1 for full model results). This suggests mean rankings for vegetarian options were 1.23 units higher than mean rankings for meat options—equivalent to each vegetarian option being ranked one place worse on average than the equivalent meat options.

### Interactions by usual meat consumption

**Fig. 1:**
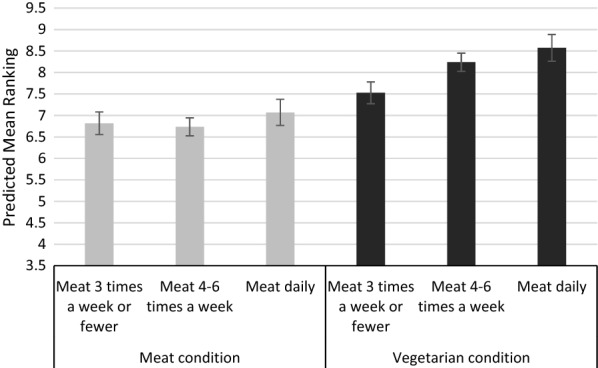
Predicted mean rankings of target options for meat vs. vegetarian target options by usual meat consumption. N.B. Error bars represent 95% CIs. Scores are based on mean ranking of items, so higher scores reflect less-selected items

Usual meat consumption and interactions between the meat vs. vegetarian condition and usual meat consumption were added into the regression model. These additions reduced the coefficient for the vegetarian condition to 0.69 (95%CIs: 0.32, 1.05)—now equivalent to the difference between conditions for those who reported eating meat 3 times a week or less. There were no significant main effects of usual meat consumption (coefficient for meat 4–6 times per week: − 0.1, 95%CIs: − 0.4, 0.3; coefficient for meat daily: 0.3, 95%CIs: − 0.1, 0.7). Figure 1 shows how higher reported consumption of meat predicted worse mean rankings in the vegetarian, but not in the meat condition (interaction coefficient for meat 4–6 times per week × vegetarian: 0.8, 95%CIs: 0.3, 1.3; interaction coefficient for meat daily × vegetarian: 0.8, 95%CIs: 0.2, 1.4) (see Additional file 1: Table S2 for full model results).

## Discussion

The results of this study suggest that meat meal options are selected more often than vegetarian options even when visual attractiveness and meal type are held constant. For meat-based target options, there was overlap between the rankings for these options and comparator options. In contrast, no vegetarian option was better ranked than any of the comparator options. Meat options were increasingly selected with increasing frequency of usual meat consumption. This may reflect previous work suggesting that there may be a group of individuals characterised by high preferences for meat and who are less open to reducing their meat consumption [5].

Given the role preferences may play in guiding food selections, this pattern of results raises some concern in that preferences for meat over vegetarian options could limit the effectiveness of attempts to reduce meat consumption in our diets. Stronger interventions may be needed to counteract the impact of preferences, or interventions that could mitigate against this effect could be prioritised. For example, meat-based options were by no means always selected over vegetarian ones, and with an increased number of vegetarian options (and reduced number of meat-based options), the chances of their being a preferable vegetarian option should increase—and lead to increase selections of vegetarian options, as found in one field study [9].

In conclusion, this study suggested meat options were preferred to equivalent vegetarian options. This highlights some of the difficulties faced in aiming to make diets healthier and more sustainable by reducing meat consumption.

## Limitations

This was a relatively small study, examining a limited number of main meal options, and conducted online so that participants only had visual cues and did not receive any of the meals they selected. Nevertheless, it offers an initial exploration of the differences in preferences between vegetarian and main meals in a controlled setting, using a sample of photographs taken from cafeterias. Further research can explore a greater range of options—including whether there are differences in preferences depending on the kinds of vegetarian options available—e.g., with the primary component being vegetables, cheese, or meat substitutes, or comparing higher energy meat vs. vegetarian options. These are likely to be subject to different barriers to acceptance, e.g. meat substitutes may be subject to greater neophobia. This research could help to establish how best to implement interventions to try to reduce meat consumption—for example, by guiding implementers towards kinds of vegetarian options that might be best accepted—albeit with the health and environmental benefits of these alternatives also varying. In addition, further work could explore different ways of presenting vegetarian options to make them more attractive—for example, taking advantage of their natural colourfulness [10].

## Supplementary Information

**Additional file 1**: **Table S1 **Tobit regression model predicting the mean ranking score for target options.** Table S2 **Tobit regression model predicting the mean ranking score for target options, with interactions by usual meat consumption

## Data Availability

The dataset generated during the current study are available in the Open Science Framework https://osf.io/hcxz3/.

## Abbreviation

GCSE: General Certificate of Secondary Education
