# Supplementary material for: Are meat options preferred to comparable vegetarian options? An experimental study
Source: BMC Res Notes. 2021 Jan 26;14:37. doi: 10.1186/s13104-021-05451-9 (PMC7836153; doi:10.1186/s13104-021-05451-9)
Supplement: Supplementary file 1 — Additional file 1: Table S1 Tobit regression model predicting the mean ranking score for target options. Table S2 Tobit regression model predicting the mean ranking score for target options, with interactions by usual meat consumption [file 13104_2021_5451_MOESM1_ESM.docx]

**Are meat options preferred to comparable vegetarian options? An experimental study**

**Supplementary materials**

**Table S1.** Tobit regression model predicting the mean ranking score for target options

|  | **Coefficient** | **95% CIs** | | **p-value** |
| --- | --- | --- | --- | --- |
|  |  | *Lower* | *Upper* |  |
| Vegetarian (Ref: Meat) | 1.23 | 1.02 | 1.44 | <0.001 |
| Age | 0.002 | -0.005 | 0.008 | 0.554 |
| Female (Ref: Male) | -0.05 | -0.26 | 0.17 | 0.659 |
| Lower education  (Ref: Higher education) | 0.07 | -0.14 | 0.28 | 0.524 |
| Hunger | -0.09 | -0.16 | -0.01 | 0.030 |
| Constant | 6.80 | 6.39 | 7.20 | <0.001 |
| Sigma | 1.52 | 1.34 | 1.72 | - |

*540 observations, 0 left-censored, 36 right-censored, pseudo r-squared=0.0694*

**Table S2.** Tobit regression model predicting the mean ranking score for target options, with interactions by usual meat consumption

|  | **Coefficient** | **95% CIs** | | **p-value** |
| --- | --- | --- | --- | --- |
|  |  | **Lower** | **Upper** |  |
| Vegetarian (Ref: Meat) | 0.69 | 0.32 | 1.05 | <0.001 |
| Usual meat consumption: 4-6 times a week  (Ref: Less than 4 times a week) | -0.08 | -0.41 | 0.26 | 0.653 |
| Usual meat consumption: Daily (Ref: Less than 4 times a week) | 0.30 | -0.10 | 0.70 | 0.145 |
| Vegetarian * Usual meat consumption: 4-6 times a week | 0.80 | 0.33 | 1.30 | 0.001 |
| Vegetarian * Usual meat consumption: Daily | 0.79 | 0.22 | 1.36 | 0.007 |
| Age | 0.003 | -0.003 | 0.010 | 0.308 |
| Female (Ref: Male) | -0.01 | -0.23 | 0.20 | 0.892 |
| Lower education  (Ref: Higher education) | 0.06 | -0.14 | 0.27 | 0.551 |
| Hunger | -0.10 | -0.17 | -0.02 | 0.011 |
| Constant | 6.70 | 6.24 | 7.15 | <0.001 |
| Sigma | 1.43 | 1.26 | 1.62 | - |

*540 observations, 0 left-censored, 36 right-censored, pseudo r-squared=0.0884*

*N.B. Comparing BIC values suggests this is a better model than the one in Table S1 above – BIC of 1756.9 for this model, compared to 1766.7 for the model in Table S1*
